# Validation of multisource electronic health record data: an application to blood transfusion data

**DOI:** 10.1186/s12911-017-0504-7

**Published:** 2017-07-14

**Authors:** Loan R. van Hoeven, Martine C. de Bruijne, Peter F. Kemper, Maria M.W. Koopman, Jan M.M. Rondeel, Anja Leyte, Hendrik Koffijberg, Mart P. Janssen, Kit C.B. Roes

**Affiliations:** 1 Julius Center for Health Sciences and Primary Care, University Medical Center Utrecht, Universiteitsweg 100, 3508 GA Utrecht, The Netherlands; 2 Transfusion Technology Assessment Department, Sanquin Research, Plesmanlaan 125, 1066 CX Amsterdam, The Netherlands; 3 Department of Public and Occupational Health, EMGO Institute, VU University Medical Center, Van der Boechorststraat 7, 1081 BT Amsterdam, The Netherlands; 4 Department of Transfusion Medicine, Sanquin Blood bank, Plesmanlaan 125, 1066 CX Amsterdam, The Netherlands; 5 Isala, Dr. Van Heesweg 2, 8025 AB Zwolle, The Netherlands; 6 OLVG, Oosterpark 9, 1091 AC Amsterdam, The Netherlands; 7 Department of Health Technology & Services Research, MIRA Institute for biomedical technology and technical medicine, University of Twente, Drienerlolaan 5, 7522 NB Enschede, The Netherlands

**Keywords:** Data validation, Data quality, Routinely collected data, Linkage of multiple sources

## Abstract

**Background:**

Although data from electronic health records (EHR) are often used for research purposes, systematic validation of these data prior to their use is not standard practice. Existing validation frameworks discuss validity concepts without translating these into practical implementation steps or addressing the potential influence of linking multiple sources. Therefore we developed a practical approach for validating routinely collected data from multiple sources and to apply it to a blood transfusion data warehouse to evaluate the usability in practice.

**Methods:**

The approach consists of identifying existing validation frameworks for EHR data or linked data, selecting validity concepts from these frameworks and establishing quantifiable validity outcomes for each concept. The approach distinguishes external validation concepts (e.g. concordance with external reports, previous literature and expert feedback) and internal consistency concepts which use expected associations within the dataset itself (e.g. completeness, uniformity and plausibility). In an example case, the selected concepts were applied to a transfusion dataset and specified in more detail.

**Results:**

Application of the approach to a transfusion dataset resulted in a structured overview of data validity aspects. This allowed improvement of these aspects through further processing of the data and in some cases adjustment of the data extraction. For example, the proportion of transfused products that could not be linked to the corresponding issued products initially was 2.2% but could be improved by adjusting data extraction criteria to 0.17%.

**Conclusions:**

This stepwise approach for validating linked multisource data provides a basis for evaluating data quality and enhancing interpretation. When the process of data validation is adopted more broadly, this contributes to increased transparency and greater reliability of research based on routinely collected electronic health records.

**Electronic supplementary material:**

The online version of this article (doi:10.1186/s12911-017-0504-7) contains supplementary material, which is available to authorized users.

## Background

Electronic health databases are vastly expanding in both the amount and scope of the data available. For health care researchers it seems very attractive to utilize these data maximally [1, 2]. Unfortunately, the use of routinely collected electronic health record (EHR) data potentially leads to quality issues, resulting from the fact that the data were not registered for research purposes but rather for clinical management or financial administration. This affects the basic quality of the data for research purpose and the ability to correctly interpret these data. Therefore, it is important to validate the quality of the data before they can serve as a source for health care research aimed to change clinical practice.

Data validity has previously been described as *whether values ‘make sense’* [3]; data are considered valid if the data represent what they claim to represent [4]. In this paper we use the term *validation* to indicate the process of assessing and improving data quality. Benefits of performing data validation are that it provides guidance on strategies to improve data quality, and, by providing an overview of data quality, enables a fair appreciation and interpretation of study results [5].

Ideally a uniform, systematic method should be used to assess, report and improve data quality. However, as noted previously [3]: *There is currently little consistency or potential generalizability in the methods used to assess EHR data. [...] researchers should adopt validated, systematic methods of EHR data quality assessment*. A review of 35 empirical studies that used electronic health care data showed that 66% of the studies evaluated data accuracy, 57% data completeness, and 23% data comparability. Even if quality measures were reported, the accuracy of variables were highly variable, ranging from 45% to almost 100% [6]. Also, information about chronic and severely ill patients was more likely to be documented as compared to healthier patients [7], which in itself may be a source of bias.

Data quality assessment is especially important in studies using data from multiple sources, in order to *distinguish true variations in care from data quality problems* [8]. More sources will provide either more cases (multicenter studies) or additional information. Whereas adding more cases can be problematic for data harmonization because different sources may use different coding systems, linking additional information (for example from external data sources) requires that patients (or other entities) can be identified and linked in all sources [9]. In each linkage step, non-linking records might result in a selection of the data that is incomplete and possibly biased. However, existing validation frameworks rarely address multiple sources linkage [10]. Only the RECORD statement, a reporting checklist for observational research using routinely-collected health data [11], and a guideline for the reporting of studies involving data linkage mention that the percentage of linked records should be provided [12].

Existing EHR data quality assessment frameworks all have different approaches, with partly overlapping dimensions or components of data validity (see Additional file 1: Table S1). Fundamental dimensions that in some form occur in most frameworks are: completeness, correctness and currency [3]. Although all of these frameworks list important aspects that should be reported, it is rarely mentioned how these aspects should be verified or appraised.

In this paper we aim to further standardize the process of data validation. To this end we developed a practical approach for assessing various dimensions of EHR data quality, directed specifically at linked data from multiple providers. The approach is applied to the Dutch transfusion data warehouse [13] and explicitly shows how to assess the validity outcomes. Thereby we provide a detailed example of how this type of data can be validated systematically. We hope that this will increase awareness among researchers of the importance and benefits of structured data validation.

## Methods

### Validation approach

**Fig. 1:**
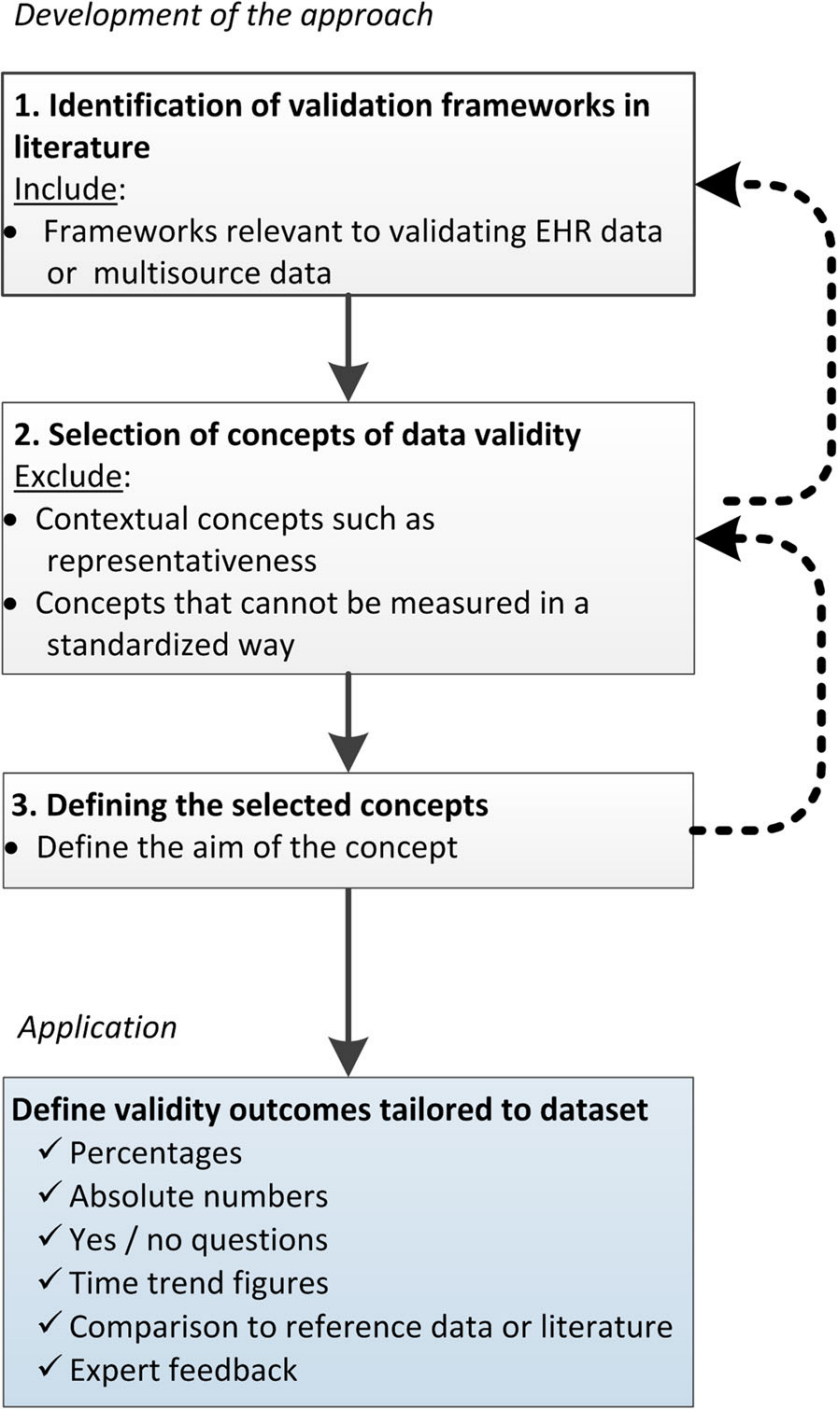
Development of the validation approach. First, validity concepts are identified, selected and defined. Second, concrete validity outcomes are established, tailored to the specific application or dataset

The validation approach starts with selecting validity concepts from previous literature and applying these to the data. First, existing frameworks were identified in the literature, from which then relevant concepts were selected, and finally, the concepts were operationalized in terms of the final application. Each of the different steps are depicted in Fig. 1 and further described below.

#### Identification of validation frameworks in literature

Previous frameworks on the validation of EHR data were identified in literature using the search terms ‘data validation’, ‘data validity’ or ‘data quality’, separately and combined with ‘electronic health record’, ‘routine patient care record’, ‘routinely collected (health) data’, ‘routine administrative healthcare data’, ‘hospital registry data’, ‘joint registry data’, ‘linked data’, ‘administrative database’, and via examining the references in those papers, until no new concepts seemed to emerge. From this literature, we selected those frameworks that might have relevance to EHR or to linked (multisource) data. In total, six data quality frameworks and two reporting guidelines were selected from literature [3, 8, 11, 12, 14–18]. Additional file 1: Table S1 provides an overview of the data quality concepts that were found in the selected frameworks and guidelines.

#### Selection of data validity concepts

From the frameworks identified, data validity concepts were selected that were applicable to validating EHR data from multiple sources. Excluded were contextual concepts that differ for each research question such as currency, timeliness, representativeness, relevance, appropriate amount of data, and accessibility; these contextual concepts might eventually be addressed at a later stage. Also excluded were concepts that can only be assessed by manually reviewing the original medical records; instead only concepts that can be assessed in a more standardized way were included.

**Fig. 2:**
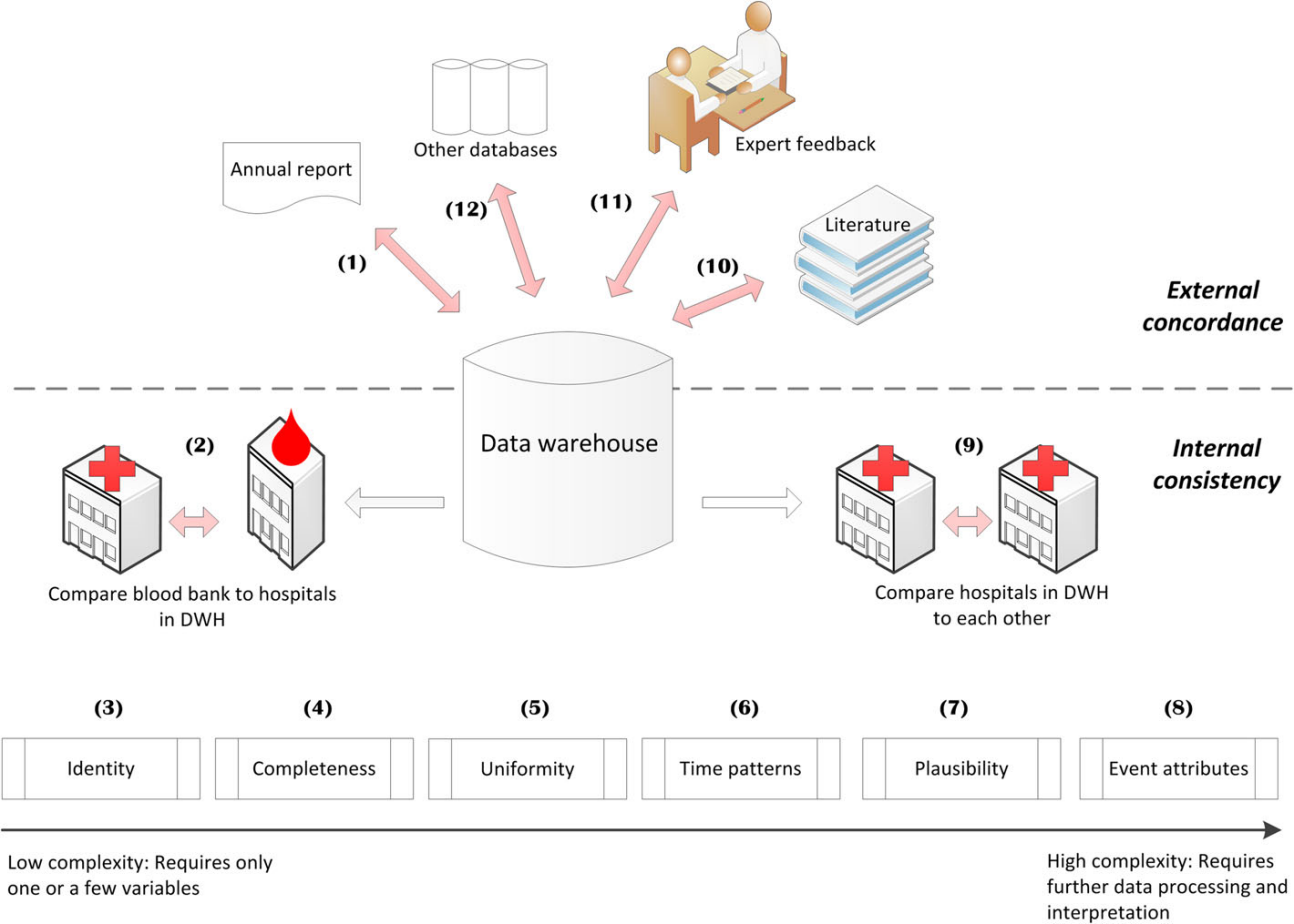
The validation approach. The approach distinguishes external validation concepts (upper part) and internal validation (lower part) concepts. The numbers indicate a suggested order in which to check to concepts in order to efficiently identify errors in the data

The following concepts were included in our approach (numbers within parentheses reflect the number of frameworks that include these concepts in some form): External concordance (3), Linkage (3), Identity (7), Completeness (3), Uniformity (4), Time patterns (2), Plausibility (6) and Event attributes (1). The concept External concordance was split into four separate concepts: External concordance with (annual) reports from related organizations, External concordance with earlier findings in literature, External concordance with external clinical registries or databases, and External concordance with expert feedback. Also, because of the multisource character of our application, we added the concept Consistency of hospitals within the data warehouse. All selected validity concepts are depicted in a step-wise approach (Fig. 2). The data warehouse is depicted in the center, with arrows leading to the concepts. Broadly, the concepts and outcomes can be categorized as either external or internal. *External concordance* (depicted in the upper part of Fig. 2) is the agreement between aggregated numbers in the data warehouse and external sources. For example, the numbers in the data warehouse can be compared to (annual) reports from related organizations. Likewise, earlier findings in literature can be used, or external clinical registries or databases might be available for comparison. Finally, the numbers and findings can be checked by presenting them to experts in the field. *Internal consistency* outcomes (depicted in the lower part of Fig. 2) use expectations of what are considered valid values, often within one data source, or valid relationships between and within variables. Internal consistency concepts are: Linkage of entities occurring in multiple data tables within the data warehouse, Identity, Completeness, Uniformity, Times patterns, Plausibility, Event attributes and Consistency of results between hospitals within the data warehouse.

#### Defining the selected concepts

For each concept selected the aim was defined, i.e. what would be perfect validity in terms of this concept (Table 2 under the column header ‘Aim’). In addition, an order is suggested in which to check the concepts that is efficient in identifying errors in the data (numbered steps 1–12 in Fig. 2). In general, one would start with concepts that are relatively easy to check, and end with concepts that require further processing of the data. Applying this general logic, the first step is to start with External concordance of the raw numbers in the data warehouse as compared to external data from for instance an annual report (Step 1 in Fig. 2). If crude numbers are incorrect a return visit to the data provider is necessary to check whether the correct data can be provided. In Step 2, it should be ensured that entities occurring in multiple data tables can be linked, and it must be decided whether records that cannot be linked will be excluded or not, before the data on the other concepts are validated. Next the application of the Identity (Step 3) and Completeness (Step 4) concepts is straightforward: The requested variables should be present, ideally have no missing values and single entities or events should be unique. If the dataset is incomplete or in case duplicates exist, this might bias the other validity outcomes. When data have no duplicates and is as complete as possible, the remaining *Internal consistency* concepts can be checked: Uniformity, Time patterns, Plausibility and Event attributes (Step 5–8). The Uniformity concept (Step 5) checks and ensures that measurements across time and departments all have the same units and/or coding system and/or duration. This is especially important for diagnoses and procedures; ideally hospitals should use similar coding systems, with the same level of detail, and use them in the same way also over time. Time patterns (Step 6) within one variable or linkage patterns between multiple variables might reveal the occurrence of registration or extraction errors through large gaps or unexplained changes that occur over time. The Plausibility concept (Step 7) examines the data on identifiable errors, using expectations of relationships between variables to check the accuracy of measurements, for instance the accuracy of date and time values. The Event attributes concept (Step 8) requires that for each event (e.g. a hospitalization or procedure) all relevant attributes are present (e.g. measurements). Finally, when validity outcomes have been computed for various centers within the data warehouse, the observed differences between hospitals from within the data warehouse can be compared (Step 9). This will either support the validity of the data when the outcomes between centers are consistent, or might indicate errors in the data or findings; unexplained differences between centers might warrant further investigation.

After the *Internal consistency* concepts have been checked and -if necessary- improved, the final *External concordance* outcomes can be computed. These outcomes require some preliminary analyses to be done on the data, and then comparing the findings to previous literature (Step 10) and discussing the results with clinical experts (Step 11). Finally, the resulting validity outcomes can be placed in context by comparing them to similar databases, if available (Step 12).

### Example case

#### Application

To apply the concepts to a specific dataset, tangible outcomes per concept need to be defined, preferably in quantifiable terms such as percentages or absolute numbers. Outcomes can also be yes/no questions, time trend figures, comparisons to reference data or literature, or expert feedback. As the outcomes are tailored to the specific application, this step of defining the exact outcomes has to be repeated for each unique dataset.

#### Data

Data on blood transfusion were used as an example case to illustrate the application of the validation approach. These data were collected in the context of the Dutch Transfusion Data warehouse (DTD) project [13], in which data from the national blood bank on blood donors and products are linked to patient data from two teaching hospitals for the period 2010–2014 (see Table 1 for hospital characteristics). Both hospitals use the national Blood transfusion Guideline [19]. Variables include the three most important blood products that are transfused: red blood cell (RBC), fresh frozen plasma (FFP) and platelet (PLT) products, as well as clinical information on transfusion recipients such as diagnoses, surgeries and laboratory measurements. After collection, these data need to be validated in order to create a valid transfusion data warehouse that can be used for research purposes. As this dataset involves multiple centers and linked donor-recipient data, it is especially suitable to serve as an example case to illustrate the validation approach. In order to keep the Results table manageable, the average outcomes of the two teaching hospitals are shown.

**Table 1:** Hospital characteristics (for the year 2014)

	Hospital A	Hospital B
Number of beds	1100	471
Annual number of RBC transfusions	12,653	6681
Presence of typical transfusion specialisms	Hematology, oncology, thoracic surgery, trauma center	Hematology, oncology, thoracic surgery, trauma center (and heavy emphasis on major vascular / aneurysm surgery and obstetrics)

## Results

The validation approach was applied to the transfusion dataset, so that each concept was assessed by one or more outcomes (Table 2). A selection of outcomes that demonstrate how the validation process led to improvements of the data is discussed in more detail below. A more extensive discussion of all validity outcomes can be found in Additional file 1: Table S2.

**Table 2:** Concepts and applied validation outcomes for *n* = 2 hospitals from the DTD

*Validation Approach*			*Application*	
Order	Concept	Aim	Outcome	Average of two hospitals
*External*				
1	Concordance with report	Data are concordant with (annual) report	% agreement between number of products in annual blood bank report and DWH	98.7% (RBC 99.2%, PLT 97.6%, FFP 98.7%)
10	Concordance with literature	Data are concordant with previous findings in literature	Comparison of distribution of blood products by age and gender per product type in the Netherlands	Distributions were quite similar, only platelet use has shifted towards younger patients (Additional file 1: Figure S2.4).
11	Concordance with experts	Data are concordant with expert opinions; findings can be explained in a clinical context	Plausibility of changes in Hb after blood transfusion	The experts concluded that the plausibility is acceptable; the 1% unexpected decreases might be explained by other factors.
12	Concordance with other databases	Findings are concordant with other databases	Comparison of findings with SCANDAT, a Scandinavian transfusion database	The SCANDAT database has similar external concordance, completeness and linkage rates.
*Internal*				
2	Linkage data sources within DWH	Entities occurring in multiple data tables can be linked	% transfusions linked to issued products by id of the end product	99.96% (no link for *n* = 46 RBC, *n* = 5 PLT, and *n* = 1 FFP)
			% products issued linked to transfusion (indicates spilling rate)	97.65% (RBC 97.95%, PLT 99.25%, FFP 93.35%)
			% products that can be linked to donation(s); % products linked to donors	Initially 96.73%, after improving the donation numbers this increased to 99.99%; the link from product to donor was 99.98%
3	Identity	No duplicates	% duplicated transfusions (donation identification code + product type)	0.14% (initially this was 1%; it turned out that most duplicates were split products. Due to unavailable product codes in one hospital, the broader product type had to be used)
			% duplicated donations (donation identification code + product code)	0.005% (RBCs); 0% (FFP and PLT)
			% duplicated procedures codes	0% (all duplicates were removed, because it was expected that double registration would occur)
4	Completeness	No missing variables or values	% patient ID; date of birth; gender, procedure date; Hb and thrombocyte counts; product code	100%; 99.99%; 99.99%; 100%; 99.8% and 97.5%; 50%
			% non-missing values for donor ID; date of birth; gender, Hb value, Expiration or Production date	100%; 99,995%; 100%; 98.8%; 100%
			% of transfusions that fall within at least one diagnosis start and end date	98% (see Additional file 1: Table S2.1)
5	Uniformity	Measures across time and data sources all have the same units, level of detail and/or coding system	% product codes that occur in the reference list of ISBT product codes	50% (for one hospital product code was not available)
			% Diagnosis codes that occur in the reference list (of national diagnosis codes and descriptions)	96.1%
			% of Hb measurements from hospitals and blood bank with the same level of precision	>99.6% uses 1 significant decimal in all sources
6	Time patterns	No unexplained changes over time	Compare number of donations, products and donors of subsequent (calendar) years	The observed decrease (Additional file 1: Figure S2.1) is in line with the known nationally decreasing trend.
			Examine number of transfusions per year per product type	The relatively high decrease for FFP use (Additional file 1: Figure S2.2) can be explained by the introduction of ROTEM, a hemostasis testing method.
			Examine linkage percentage of transfusions to products issued per year	In 2010 relatively many unlinked transfusions occurred (see Additional file 1: Figure S2.3). After blood bank data from the previous year 2009 was included, the linkage percentage increased to 99.8% or higher for all years.
7	Plausibility	Data are free of identifiable errors	% donation date < date of pooling	100%
			% within limits for number of donations per donor per year (maximum is 3 (females) or 5 (males) for whole blood and 23 for plasma)	FFP 100%; WB 99.8% (0.2% exceeds the limit with in total 6 or 7 donations within a year)
			% donor age > 18 and >70 years (minimum and maximum age for donating)	100% (only 0.0006% was >70 and 0.0004% was <18 and these were mainly autologous donations)
			% transfusion with increase (and decrease) in Hb level (Hb values + − 1 day around transfusion; difference > + − 8.8% is considered a clinical change)	54% increases; 6% decreases; 40% no change. Of those decreasing, 97% had a diagnosis indicating high bleeding risk
			% patient age < 121 years	100%
			Maximum number of transfusions per year	Max tr. per year 476 (mainly FFP) for diagnosis TTP.
			% correct gender for Gynecology diagnoses	100%
			% patients with transfusions/ surgery after date of death	0.0% (*n* = 2 changed mortality status to NA)
			% with admission date before discharge date) (zero-length rule)	100%
			% with non-negative difference between expiration and transfusion date	99.93%
8	Event attributes	All attributes relevant to an event description are present	% of pooled products that are linked to the correct number of unique donors (in this case 5 or 6 donors contribute to one pooled platelet product)	100%
			% of patients that are transferred to another hospital according to the ‘discharge destination’ variable	6%
			% transfusions linked to hospitalization (indicates outpatient transfusions)	99.16% (of which 23.64% day admissions, likely including transfusions given at the outpatient ward)
9	Consistency hospitals within DWH	No unexplained differences between hospitals	Comparison of (validity) outcomes of the hospitals	The two hospitals have very similar validity outcomes, not requiring further investigation.

We first checked the agreement between the number of blood products in the dataset and those reported in the annual blood bank report (Step 1: External concordance with report), which was 98.7%, computed as the absolute difference between both sources as the numerator (computed separately per product and summed) and the number of blood products in the dataset as the denominator. The slight disagreement can be explained by potential differences in the way of counting composite and split blood products. Of the transfused products, initially 96.7% could be linked to the corresponding donation (Step 2: Linkage). We traced this difference back to a post-hoc modification in the coding of the product identification number at the blood bank, leading to different codes existing in the blood bank and the hospital system for the same product. When the coding was adjusted, the proportion linked products increased to 99.98%. Initially 1% of products were duplicated (Step 3: Identity). Investigation of product types revealed that most duplicates were split products. The products were given unique identifiers post-hoc, resulting in an improved duplication percentage of 0.14%. Most transfusions −98%- could be linked to one or more diagnoses (Step 4: Completeness). In most cases the diagnosis was even more than complete: the number of pending diagnoses ranged up to 15 diagnoses per transfusion. This means that it will be necessary to make a selection of those diagnoses in the future if we want to determine the main indication for a transfusion. Diagnoses were defined differently by the two hospitals and therefore had to be recoded using a uniform reference table (Step 5: Uniformity). The percentage of diagnosis codes that could be linked to the reference table was 96.1%. Investigation of time patterns (Step 6: Time patterns) revealed that for the year 2010, an exceptionally high percentage of transfusions could not be linked to products issued (2.2% versus 0.07% in other years). This percentage could be lowered to 0.17% by including blood bank data from the previous year 2009 (the unlinked products were mainly frozen plasma products that were issued in the year before the actual transfusion). Plausibility of registered dates and times (Step 7: Plausibility) was checked based on a priori expectations. For example hemoglobin (Hb) generally is expected to increase after transfusion. Indeed, in 54% of cases Hb did increase, 40% did not clinically change, however 6% decreased. This decreasing 6% might indicate incorrect date values, which could occur when the registered time of transfusion actually records the moment that a product or service (e.g. the blood product) was requested instead of administered. To check this, expert feedback was asked regarding the plausibility of the observed Hb changes (Step 11: Concordance with expert feedback). Further investigation of the data showed that most recipients with a decrease in Hb had a diagnosis indicating high bleeding risk (87%), explaining the observed decrease. Taking this into account, the percentage of all transfusions with an unexplained decrease is lower than 1%, which according to the experts is acceptable. Event attributes (Step 8) include that each platelet product is made up from multiple donations and should therefore be attributable to five or six unique donations, which was also found in the dataset for 100% of platelet products.

These are all average outcomes, however a comparison of the two hospitals included shows that their validity outcomes were very similar (Step 9: Consistency of hospitals within data warehouse; results not shown), supporting the validity of the findings. Also, concordance with literature (Step 10) was checked by comparing the distribution of blood products over age and gender per product type with the previously reported PROTON study, of which the DTD is the successor. Distributions were very similar but platelet use has shifted towards older patients, especially men aged 60–80 years (Additional file 1: Table S2). This can be explained in part by the ageing of the population and changes in policy in the past 10 years; platelet use was increased in thorax surgery and hematological disorders, which both are more prevalent in men.

Lastly, the concordance of these findings with validity outcomes reported for other databases was investigated (Step 12: Concordance with other databases). The most extensive list of validation outcomes were reported by the SCANDAT study [18, 20], therefore, these outcomes are shown next to the validity outcomes of the DTD (Table 3). SCANDAT and the DTD show similar results regarding the high external concordance of the data with external statistics and the fact that both studies identified missing data by investigating time patterns. Different is the proportion of hospitalized patients, which might be due to differences between the countries in the registration of patients (we found a consistently higher hospitalization rate for both of the DTD hospitals included). The estimated proportion of patients with incomplete information due to transference to another hospital was up to 6% for the DTD. This might actually be an underestimation, considering the finding that in SCANDAT 8.9% of recipients received a blood transfusion in two or more local registers, and because our 6% did not include patients who were hospitalized elsewhere prior to being hospitalized in our included hospitals.

**Table 3:** Comparison of validity outcomes in the SCANDAT study and the current DTD results

Outcome	SCANDAT 1/2	DTD example
External concordance of database and official statistics on the number of transfusions	>97%	>98.7% for products and 99.96% for transfusions
% transfusions linked to the corresponding donor	95%	99.99%
% transfusions linked to hospitalization	88.7%	99.2% (of which 23.6% day admissions)
% duplicated donations and transfusions	4.9% (donations) and 9.1% (transfusions)	0% (donations) and 0.14% (transfusions)
% missing or invalid values for identification number or date values	Range between 0.1% to 3.6%	0%–0.01%
Time patterns for donations and transfusion counts	In 1 year approximately 160,000 transfusions were missing; it took 2 years for the number of donations and transfusions to stabilize after the start of a new registration system	In 1 year the link of transfusions to products could be made for 2.2%, however this could be improved by adding donation data from the previous year
% of recipients had records of receiving a blood transfusion in two or more local registers	8.9%	6% of patients are transferred to another hospital

## Discussion

Being explicit about research methods is important for the reliability, reproducibility and credibility of research, and in our opinion this includes being explicit about data validity. We recommend that any study that involves electronic health record data should include an overview of the steps taken to ensure data validity. This applies in particular to more complex routinely registered data that are not designed for research purposes or are complex (for example data covering an extensive time period or linkage of several sources). Therefore, we documented a step-wise approach for systematically validating multiple source data from electronic health records. The approach integrates concepts from existing guidelines for EHR quality assessment with the specific challenges inherent to linked, multisource data. The proposed approach is practical as the validity concepts are directly related to what is needed for actually carrying out the validation. For some validation steps, data from only one center and of a single point in time might be sufficient, other steps require at least two centers or data from prolonged periods of time, or require the availability of external information such as previous literature or expert opinion.

The approach was applied to data from the Dutch Transfusion Data warehouse (DTD), resulting in an overview of validity outcomes and improvements of the quality of the data. In addition to improving the data, the validity outcomes, if made publicly available, increase transparency and contributes to the efficient use and reuse of existing sources of information. A clear overview of data validity is informative for researchers who want to use an existing database and, vice versa, requests for using the data can be evaluated more easily.

### What is ‘good’ quality data?

It can be argued that no universal cut-off values exist as to whether electronic health data are “valid” or of “high quality” [21], since the level of data quality required will also depend on the purpose of the study concerned. Still, objective measures for relevant quality concepts are necessary, and an adequate understanding of how to interpret validity outcomes is desirable.

Levels of validity as found in earlier studies might set a -more or less arbitrary but realistic- standard. Validation outcomes reported by previous transfusion data warehouse studies are sparse and vary greatly. Most often reported was the linkage rate of transfusions to donors, varying between 92%–99% [20, 22–25] and, vice versa, estimates of wastage of blood products (i.e., issued but not transfused) of 1.3% and 7.7% [22, 23]. The percentage missing values was also reported by some studies: clinical variables were missing for 13% (post-transfusion Hb), 14% (ASA code) [26], and 20% (specialty), with the degree of missingness varying between specialties from 2% to 47% [21] (a more extensive overview per transfusion database is provided in Additional file 1: Table S3). Comparing the SCANDAT to the DTD outcomes, we found similar results regarding the completeness measures and the high external concordance of the data with external statistics. In this context, we think the data from the DTD shows sufficient validity. This is, however, time-bound; when new data (either hospital or external) are included in the future, these data must be validated as well.

Although data quality should ideally be checked continuously, such checks may be particularly relevant following: the first data extraction from a hospital or other data source, inconsistencies present in the data, the introduction of a new hospital information system, or reorganization or fusion of hospitals [1]. In order to keep track of changing registration systems in individual hospitals, a questionnaire might be submitted once a year on new developments in the hospital that could impact the registration or data extraction. Especially for the purpose of benchmarking hospitals (or other data sources), it is important that all selection and interpretation steps are performed in a similar way for each hospital. After all, the impact on the comparison is minimized when bias is similar for each hospital.

### Remaining issues and future directions

The concept Accuracy or Correctness -which is particularly relevant for diagnosis and procedure codes- was not yet covered by the approach. As the data warehouse is too large to manually check accuracy of diagnoses for each individual patient, a sample of diagnoses and procedures will be validated by manual review of patients’ local medical records.

It must be noted that it is a choice whether to aggregate the validation outcomes to the level of patients, variables, hospitals or even combining all sources in the database. Outcomes become less informative for higher aggregation levels, but are still useful for detecting large irregularities in the data. To simplify the interpretation of validity outcomes, we encourage adding visualizations or summaries (especially when outcomes are similar), for example, Completeness could be summarized by giving the percentage of variables that is at least 95% complete [14]. In the same respect, linkage can be performed across different levels. In this paper linkage took place on the level of the patient, donor and transfusion/blood product. Different linkage scenarios include EHR data with insurance claims, mortality or medical practitioner data. The linkage percentages should be considered from the viewpoint of the particular research concerned and the impact of low linkage quality on study outcomes.

It appears that the process of preparing data for analysis, including data harmonization and assessing data quality, is commonly taking place at the intersection of data management and research. In practice, part of the validation may be performed by the data manager. However, it is the researcher who ultimately is responsible for making and communicating any choices made in this process, and the implications for the validity and interpretability of study results.

## Conclusions

The proposed approach provides a structure for validating multisource EHR data. By making the validation steps explicit and concrete, the applied example shows that the approach is feasible, enhances the interpretation of the data and improves data quality. Hopefully this will encourage researchers to consider and report data quality, in a way that goes beyond conceptual classifications and allows transparent assessment of the potential impact of data quality on research findings.

## Additional file

Additional file 1: Table S1. Occurrence of data quality concepts in existing EHR quality assessment or data linkage frameworks. **Table S2.1.** Distribution of number of pending diagnoses per transfusion. **Figure S2.1.** Time patterns in number of donations, products and donors. **Figure S2.2.** Time patterns in number of transfusions by product type. **Figure S2.3.** Time patterns in % of transfusion that initially could not be linked to products issued. **Figure S2.4.** Comparison with previous literature: Distribution of blood products over age and gender, by product type. **Table S3.** Data validity outcomes reported in the literature for studies that use transfusion databases. Text. Similarities with other operationalizations of data validity. (DOCX 205 kb)

## Abbreviations

DWH: Data warehouse
EHR: Electronic health record
FFP: Fresh frozen plasma product
Hb: Hemoglobin
ICD: International classification of diseases
PLT: Platelet product
RBC: Red blood cell product
