# Supplementary material for: Validation of multisource electronic health record data: an application to blood transfusion data
Source: BMC Med Inform Decis Mak. 2017 Jul 14;17:107. doi: 10.1186/s12911-017-0504-7 (PMC5512751; doi:10.1186/s12911-017-0504-7)
Supplement: Additional file 1: Table S1. — Occurrence of data quality concepts in existing EHR quality assessment or data linkage frameworks. Table S2.1. Distribution of number of pending diagnoses per transfusion. Figure S2.1. Time patterns in number of donations, products and donors. Figure S2.2. Time patterns in number of transfusions by product type. Figure S2.3. Time patterns in % of transfusion that initially could not be linked to products issued. Figure S2.4. Comparison with previous literature: Distribution of blood products over age and gender, by product type. Table S3. Data validity outcomes reported in the literature for studies that use transfusion databases. Text. Similarities with other operationalizations of data validity. (DOCX 205 kb) [file 12911_2017_504_MOESM1_ESM.docx]

AddiTIONAL file 1

Table S1 Occurrence of data quality concepts in existing EHR quality assessment or data linkage frameworks.

| **Authors (year)** | **Name / Scope** | **External concordance** | **Linkage** | **Identity** | **Completeness** | **Uniformity** | **Time patterns** | **Plausibility** | **Event attributes** |
| --- | --- | --- | --- | --- | --- | --- | --- | --- | --- |
| Wang & Strong (1996) | Data quality assessment |  |  |  | x |  |  | x |  |
| Kahn et al. (2012) modified from Maydanchik (2007) | Single-site and multisite EHR data quality |  | x | x | x | x | x | x | x |
| Van den Broeck, Cunningham, Eeckels and Herbst (2005) | Data cleaning |  |  | x | x |  | x | x |  |
| AHIMA (2012) | Data quality management model |  |  | x |  | x |  | x |  |
| Bohensky et al. (2011) | Guideline for the reporting of studies involving data linkage |  | x | x |  | x |  |  |  |
| Black & Payne (2003) | Checklist data quality | x |  | x |  |  |  | x |  |
| Weiskopf & Weng (2013) | EHR data quality assessment | x |  | x |  |  |  | x |  |
| Benchimol (2015) | Reporting of studies using observational routinely-collected health data (RECORD checklist) | x | x | x |  | x |  |  |  |

Supplementary file 2

### Concordance with (annual) report

The agreement between the numbers found in the database and the annual report of the Blood bank was >98.7% for number of products (varying slightly for the different product types) and 99.96% for number of transfusions.

### Linkage of data sources within data warehouse

Linkage of transfused products to products issued by the blood bank was possible for 99.96% of all transfusions, using the identification number of the end product. Vice versa, 97.65% of products issued could be linked to actual hospital transfusions (indicating the spilling rate). Initially, only 96.727% of the products could be linked to their donation(s). We traced this back to a post-hoc modification in the coding of the product identification number at the blood bank, leading to different codes existing in the blood bank and the hospital system for the same product. When the coding was adjusted, the proportion linked products increased to 99.996%.

### Identity

Every blood product should be uniquely identified by the combination of the donation code and the product code. In the blood bank data, a small percentage of duplicated products was found for RBC products of 0.005%; for FFP and PLT this was 0%. For one hospital, product codes were not available, therefore the broader product type was used. Based on donation code and product type, initially 1.00% of products were duplicated. It turned out that most (71.7%) of these duplicates were split products, which explains why the donation code and product type were similar. A potential pitfall is the double registration of events, for example multiple procedures that actually occurred must be differentiated from duplications in procedures registered for another purpose (e.g. financial registration). Therefore duplicated procedures (i.e. within the same patients at the same time) were removed, resulting in 0% duplicated procedures.

### Completeness

Most important variables are present and non-missing: for the blood bank data (donor identification code, date of birth, gender, hemoglobin value, product expiration or production date) completeness of at least 98.8% and for hospital data (patient identification code, date of birth and gender) at least 99.99% completeness.

For the outcome regarding completeness of diagnoses, in Table S2.1 the distribution of number of pending diagnoses per transfusion is shown to range from 0 diagnoses to up to 15. The percentage of transfusions that fall within the start and end date of at least one diagnosis was 98%. This implicates that it will be necessary to make a selection of those diagnoses in the future if we want to determine the main indication for a transfusion.

### Uniformity

Diagnoses and procedures were recoded into a uniform system, resulting in a linkage percentage of diagnosis codes with the reference table of 96.1%. Hb level was was registered with the precision of 1 significant decimal for >98.6% for the hospitals and 99.76% for the blood bank data.

### Time patterns

The time patterns in number of donations, products and donors (Figure S2.1) reveal no unexpected trends, as the observed decrease is in line with the known nationally decreasing trend in RBC use. The trends in blood use by product type confirm this and also show a high relative decrease for FFP products, for example from 2010 to 2011 (Figure S2.2). This decrease in FFP can be explained by the introduction of ROTEM (a method of hemostasis testing in whole blood) for thoracic surgery, and different guidelines and consensus. In the time period concerned, use of ROTEM followed a reverse trend, increasing where FFP use decreased.

Figure S2.3 shows that in 2010, the percentage of transfused products that could not be linked to products issued was exceptionally high (2.2% versus 0.07% in other years). This percentage could be lowered to 0.17% by including blood bank data from the previous year 2009 (the unlinked products were mainly frozen plasma products that were issued in the year before). This resulted in an annual linkage percentage of 99.8% or higher.

### Plausibility

Accurate date and time values are crucial in order to study cause-effect relationships, such as transfusion triggers and pre and post transfusion targets. A problem occurs if the registered (e.g. transfusion) time actually records the moment that a product or service (e.g. the blood product) was requested instead of administered. Generally, hemoglobin (Hb) should increase after transfusion. To check this, the difference was computed between the last Hb before and first Hb after transfusion (only Hb measurements within one day before or after the transfusion were considered). A clinically significant Hb change was defined as an increase or decrease of 8.8% relative to the first Hb measurement. This cut-off point was defined using the formula for the critical change: 2.77 * √ (CVa^2^ + CVi^2^) [27]. Assuming an analytical variation (CVa) of 1.5% and an intra-individual biological variation (CVi) of 2.8% [28], the cut-off value lies at 8.8%. Although we would expect that Hb increases after transfusion, it turned out that 40% did not clinically change and 6% even decreased. Recipients with a decrease in Hb were further examined and 87% of these patients had a diagnosis indicating high bleeding risk such as the diagnosis acute bleeding, justifying the validity of a decreasing Hb value.

### Event attributes

At the blood bank, blood products made from platelets (PLT) are produced by pooling the PLT of five donations and one FFP unit together. These pooled products should therefore be linked to five or six donations, which was the case for 100%.

Another attribute of a transfusion event is a hospitalization; it was found that 99.16% of all patients were also admitted (of which 23.64% were day admissions), indicating that the remaining transfusions were given in an outpatient ward. Finally, an estimate of the proportion of potentially missing information on for example transfusions was given by the percentage of patients that were transferred to another hospital. According to the 'discharge destination' variable, 6% of patients were, at the end of their admission, sent to another hospital.

### Consistency of hospitals within data warehouse

The validity outcomes for both hospitals were expected to be quite similar as both are teaching hospitals. The two hospitals had indeed very similar outcomes, supporting the validity of the findings.

### Concordance with literature

Comparison of our data with previous literature on the distribution of blood products over age and gender categorized by product type [29] revealed that the distributions were quite similar, but that platelet use has shifted towards older patients (Figure S2.4). This can be explained in part by the ageing of the population, but also by changes in policy in the past ten years: thorax surgery has increased its platelet use, and also treatment of haematological disorders has become more intensive, including higher platelet use. As both heart disease and haematological disorders are more prevalent in men, there is a peak in platelet use for men aged 60-80 years. Because relatively more platelets are transfused to older patients, platelet use for children makes up a smaller part of the total use.

### Concordance with expert feedback

Expert feedback was asked regarding the accuracy of Hb measurements. The outcomes of Step 7 (Plausibility) were presented to two clinical chemists from the participating hospitals, in order to evaluate whether these numbers seem plausible. The experts concluded that the percentage with an unexplained decrease is below 1% of all transfusions, which is acceptable. The finding that in patients with high bleeding risk, Hb value sometimes decreases and sometimes increases is also plausible; with acute bleeding it is more difficult to measure the Hb, which might lead to too much blood being given.

### Concordance with other databases

What previous transfusion data warehouse studies have reported in terms of data validation varies greatly (a more extensive overview per transfusion database is provided in Supplementary file 3). The most extensive list of validation outcomes were reported by the SCANDAT study [18,19], therefore, these outcomes are shown next to the validity outcomes of the DTD (Table 3). SCANDAT and DTD show similar results regarding the high external concordance of the data with external statistics and the fact that both studies identified missing data by investigating time patterns. Different is the proportion of hospitalized patients, which might be due to different registration of patients between the countries (as we found a consistently higher hospitalization rate for both of the DTD hospitals included). The estimated proportion of patients with incomplete information due to transference from our hospitals included to another hospital was up to 6% for the DTD. This might actually be an underestimation, because this 6% does not include patients who were hospitalized elsewhere prior to being hospitalized in hospitals analyzed, and given the findings that in SCANDAT 8.9% of recipients received a blood transfusion in two or more local registers.

Other transfusion database studies reported only a few outcomes: the linkage rate of transfusions to donors between 92%-99% [18,19,20,21,22] and, vice versa, estimates of wastage of blood products (i.e., issued but not transfused) of 1.3% and 7.7% [19,20]. The percentage missing values was also reported by some studies: clinical variables were missing for 13% (post-transfusion Hb) [23], 14% (ASA code) [22] and 20% (specialty)[23], the latter interestingly varying between specialties from 2% to 47%.

Table S2.1. Distribution of number of pending diagnoses per transfusion

| **No.**  **diagnoses** | **0** | **1** | **2** | **3** | **4** | **5** | **6** | **7** | **8** | **9** | **>=10** |
| --- | --- | --- | --- | --- | --- | --- | --- | --- | --- | --- | --- |
| **%** | 2.1 | 18.5 | 31.8 | 21.0 | 12.8 | 6.5 | 3.8 | 2.1 | 0.9 | 0.5 | 0.4 |


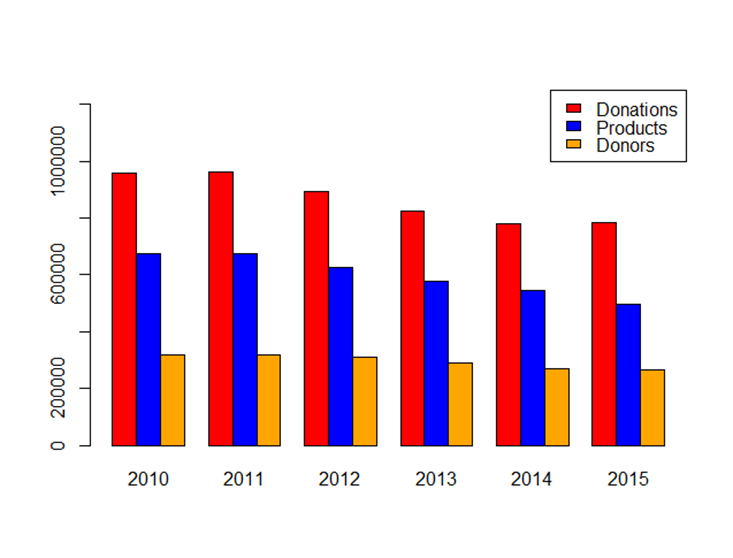


Figure S2.1. Time patterns in number of donations, products and donors


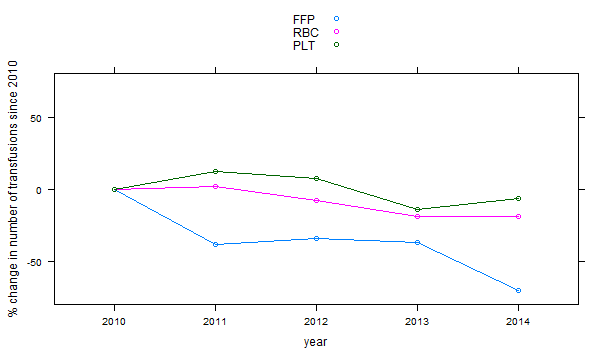


Figure S2.2. Time patterns in number of transfusions by product type


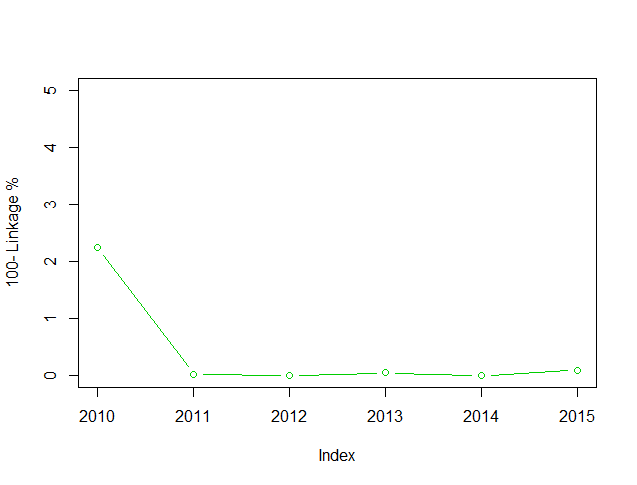


Figure S2.3. Time patterns in % of transfusion that initially could not be linked to products issued


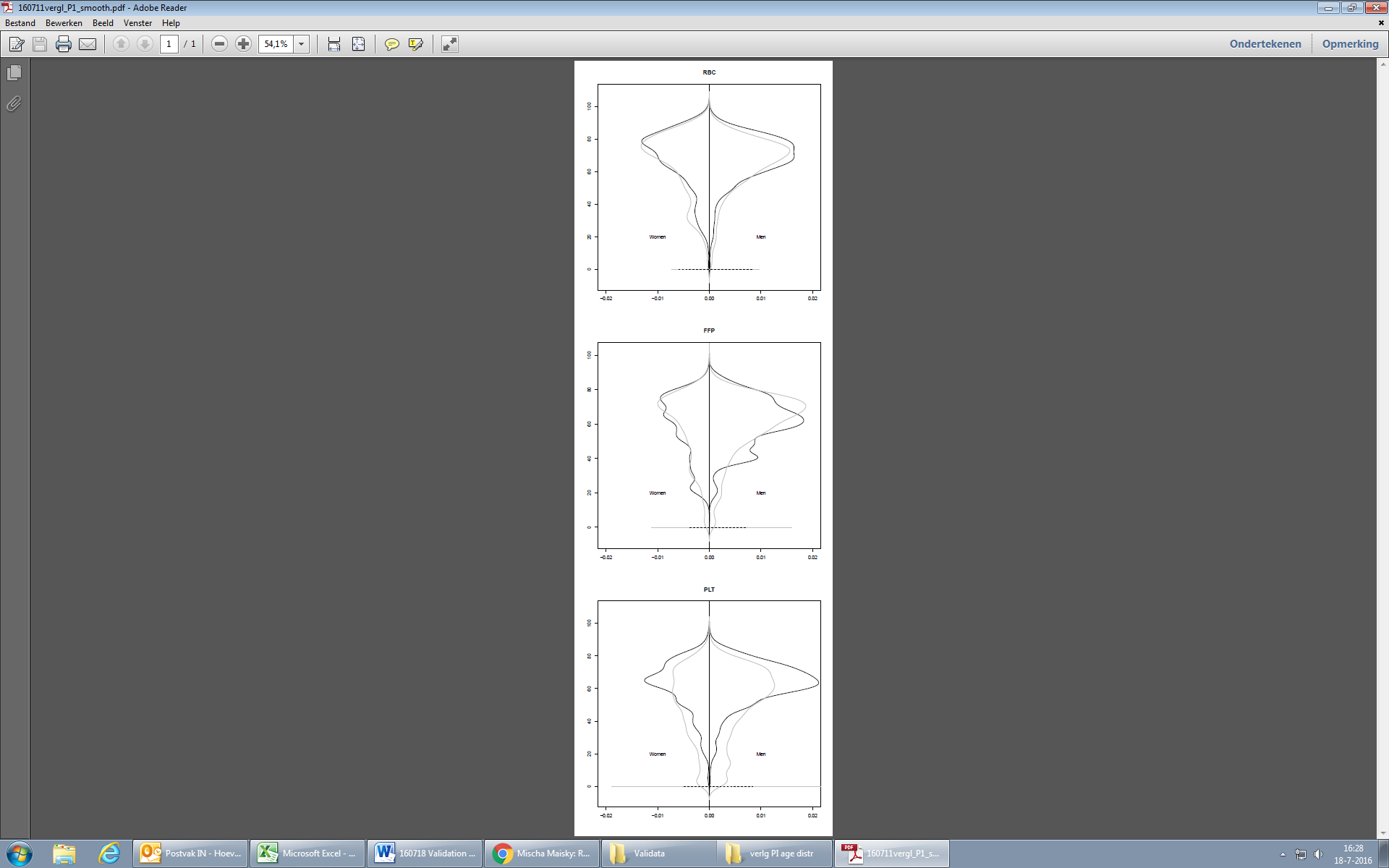


Figure S2.4. Comparison with previous literature: Distribution of blood products over age and gender, by product type. Gray line: previous study on Dutch transfusion recipients, black line: more recent data from the Dutch Transfusion Data warehouse

Supplementary file 3

Table S3 Data validity outcomes reported in the literature for studies that use transfusion databases

| **Author (Study)** | **Year** | **Concordance between number of transfusions and donations or products issued** | **Completeness** | **Identity** | **Time trends** | **Accuracy** | **Representativeness** |
| --- | --- | --- | --- | --- | --- | --- | --- |
| Edgren (SCANDAT; Denmark and Sweden) | 2006 | 92.3% | 88.7% of tr. linked to admission | Duplicates: 4.9% donor records and 9.1% transfusion records | Number of products per year was stable two years after start new system, indicating invalid first years | Invalid values: 0.5% of donor IDs, 4.5% of recipient IDs, 0.1% donation dates and 1.9% transfusion dates | Population database |
| Borkent-Raven et al. (PROTON: Netherlands) | 2010 | Not reported | 87% of transfusions linked to diagnosis | Not reported | Not reported | Not reported | Complete and incomplete records were similar to with respect to age, gender and product type |
| Barr et al. (Nothern Ireland) | 2010 | Not reported | Missing values varying between specialties (2-47%) | Not reported | Not reported | Detailed review of medical records for assignment of primary condition | Not reported |
| Allden et al. (South of Australia) | 2011 | 92.6% (reported fallout due to missing registration in the emergency department) | Not reported | Not reported | Trend in SA consistent with national trend after hospital activity weighting | Not reported | Population (SA) |
| Tinegate et al. (North of England) | 2012 | Estimated 99% | Not reported | Not reported | Change in RBC use in sample consistent with national trend | Not reported | Reported underrepresenting of hemoglobinopathies and thalassemias patients |
| Palo et al. (Finland) | 2013 | 96.8% | ASA code complete for 86% of procedures | Not reported | Data was compared between the study years (Results not provided) | Not reported | Not reported |
| Edgren et al. (SCANDAT2: Denmark and Sweden) | 2015 | 96% | Missing identification numbers for 0.5% of donations and 3.6% of transfusions | Duplicates were removed, but no % reported | Gaps over time are identified, explanations are provided. | Not reported. | Population, binational database |
| Chassé et al. (Canada) | 2015 | Not reported | Not reported | Not reported | Not reported | Random manual chart abstraction (for ICD-10CA comorbidity codes) | Population database (except one Province) |

# Supplementary file 4

## Similarities with other operationalizations of data validity

Data validity is often seen as a component of data quality, where quality consists of accuracy (including data validation) and coverage^19^, or accuracy (validity and reliability) and completeness (representativeness and comprehensiveness).^1^ We considered data to be valid if the data value measures what it claims to measure. Our Framework implicitly includes common concepts that are used when operationalizing validity: face validity, content validity, construct validity, and criterion validity.^4^ Face validity, which refers to the extent to which, subjectively viewed, a measure measures what it is intended to measure, is equivalent to External concordance with expert opinions. Construct validity describes the extent to which a value correlates with another value that aims to measure the same underlying construct, and is also measured by comparing different data sources to each other. Content validity represents the extent to which a measure samples the relevant sub-concepts of the construct. This can be seen as the representativeness of the outcomes for the concepts they represent. Criterion validity reflects the extent to which an indicator shows agreement with a gold standard of the measured domain in quality of care. When no gold standard exists, the common approach to measure criterion validity is to use construct validity.
